# Assessment and management of disease burden and quality of life in patients with hereditary angioedema: a consensus report

**DOI:** 10.1186/s13223-021-00537-2

**Published:** 2021-04-19

**Authors:** Konrad Bork, John T. Anderson, Teresa Caballero, Timothy Craig, Douglas T. Johnston, H. Henry Li, Hilary J. Longhurst, Cristine Radojicic, Marc A. Riedl

**Affiliations:** 1 Department of Dermatology, University Medical Center, Johannes Gutenberg University, Langenbeckstraße 1, 55131 Mainz, Germany; 2 Clinical Research Center of Alabama, 504 Brookwood Boulevard, Suite 250, Birmingham, AL 35209 USA; 3 Allergy Department, Hospital La Paz Institute for Health Research (IdiPaz), Biomedical Research Network on Rare Diseases (CIBERER, U754), Paseo Castellana 261, 28406 Madrid, Spain; 4 Department of Medicine and Pediatrics, Penn State University, 200 Campus Drive, Suite 1300, Entrance 4, Hershey, University Park, PA 17033 USA; 5 Asthma and Allergy Specialists, 8405 Providence Road, Suite 300, Charlotte, NC 28277 USA; 6 Institute for Asthma and Allergy, 2 Wisconsin Circle, Suite 250, Chevy Chase, MD 20815 USA; 7 Addenbrookes Hospital, Cambridge Universities NHS Foundation Trust, Cambridge and University College Hospital London, Cambridge, CB2 0QQ UK; 8 Division of Allergy and Clinical Immunology, Department of Medicine, Duke University, 1821 Hillandale Rd, Durham, NC 27705 USA; 9 University of California San Diego, 8899 University Center Ln, San Diego, CA 92122 USA

**Keywords:** Consensus document, Disease burden, Hereditary angioedema, Management, Quality of life

## Abstract

**Background:**

Hereditary angioedema (HAE) is a rare disease characterized by unpredictable, potentially life-threatening attacks, resulting in significant physical and emotional burdens for patients and families. To optimize care for patients with HAE, an individualized management plan should be considered in partnership with the physician, requiring comprehensive assessment of the patient’s frequency and severity of attacks, disease burden, and therapeutic control. Although several guidelines and consensus papers have been published concerning the diagnosis and treatment of HAE, there has been limited specific clinical guidance on the assessment of disease burden and quality of life (QoL) in this patient population. Practical guidance is critical in supporting effective long-term clinical management of HAE and improving patient outcomes. The objective of this review is to provide evidence-based guidelines for an individualized assessment of disease burden and QoL in patients with HAE.

**Methods:**

A consensus meeting was held on February 29, 2020, consisting of 9 HAE experts from the United States and Europe with extensive clinical experience in the treatment of HAE. Consensus statements were developed based on a preliminary literature review and discussions from the consensus meeting.

**Results:**

Final statements reflect the consensus of the expert panel and include the assessment of attack severity, evaluation of disease burden, and long-term clinical management of HAE caused by C1-esterase inhibitor deficiency. Patient-reported outcome measures for assessing HAE attack severity and frequency are available and valuable tools; however, attack frequency and severity are insufficient markers of disease severity unless they are evaluated in the broader context of the effect on an individual patient’s QoL. QoL assessments should be individualized for each patient and minimally, they should address the interference of HAE with work, school, social, family, and physical activity, along with access to and burden of HAE treatment. Advances in HAE therapies offer the opportunity for comprehensive, individualized treatment plans, allowing patients to achieve minimal attack burden with reduced disease and treatment burden.

**Conclusion:**

This consensus report builds on existing guidelines by expanding the assessment of disease burden and QoL measures for patients with HAE.

## Background

Hereditary angioedema (HAE) is a rare, genetic disease characterized by recurrent, unpredictable, episodes of subcutaneous (SC) or mucosal angioedema [1, 2]. The two main types of HAE are caused by mutations in the *SERPING1* gene, resulting in quantitative or functional deficiencies in C1-esterase inhibitor (C1-INH) [3]. Although most cases of HAE with C1-INH deficiency (HAE-C1-INH) are a result of autosomal dominant inheritance, 25% of cases are thought to result from de novo mutations in patients with no family history [1]. HAE-C1-INH is estimated to affect approximately 1:50,000 people, with no apparent differences due to sex or ethnicity [4]. While less common than HAE-C1-INH, a third form of HAE has also been identified in patients with a similar clinical phenotype, in which C1-INH protein levels and function are normal [5]. In some instances, HAE with normal C1-INH is associated with mutations in factor XII *(F12),* plasminogen (*PLG*), angiopoietin (*ANGPT1*), kininogen (*KNG1*), or myoferlin (*MYOF*) genes; however, in many cases the genetic cause of HAE with normal C1-INH is unknown [6–11]. This consensus focuses on the assessment of disease burden and management of patients with HAE-C1-INH, but not HAE with normal C1-INH.

HAE attacks most commonly affect the skin, gastrointestinal tract, and upper respiratory tract [4]. Attacks involving the upper airways are potentially life-threatening due to the risk of rapid-onset respiratory obstruction and asphyxiation [12]. If untreated, HAE attacks can gradually worsen over the first 12–36 h and then subside over 2–5 days [2, 13, 14]. The frequency of HAE attacks is highly variable among patients and over time [15, 16]. On average, untreated patients experience an attack every 2 weeks, with frequencies ranging from very rare to every 3 days [2, 16]. The potentially painful and debilitating symptoms of attacks may interfere with patients’ ability to conduct daily activities such as attending work or school or participating in leisure activities [13, 17, 18]. Additionally, the unpredictable nature of attacks, potential for asphyxiation, and possibility of passing the disease on to future generations result in higher levels of depression and anxiety among patients with HAE [19, 20]. Together, these factors contribute to a significant disease burden with reduced quality of life (QoL) [16, 21–27].

Therapeutic approaches for HAE include both acute and prophylactic treatments [28–31]. The goal of acute treatment is to minimize HAE symptoms during an attack, while prophylaxis aims to reduce the likelihood of swelling during an expected trigger (short-term prophylaxis) or reduce the overall recurrence of angioedema attacks (long-term prophylaxis) [4, 29, 30]. In the past decade, several targeted therapies for HAE have been developed with improved benefit-risk profiles and different treatment properties allowing for an individualized treatment approach [32–36].

Advances in acute and prophylactic treatments have resulted in a shift in HAE management from focusing on counting and treating acute attacks to developing personalized management plans with the goal of improving patient outcomes and QoL. Therefore, it is important for physicians to evaluate attack severity, assess disease burden, and optimize long-term clinical management. This consensus report aims to review updates to best practices in the management of HAE based on the availability of new therapies by evaluating existing patient-reported outcomes (PROs) and QoL measures and by providing practical guidance for a broad clinical audience.

## Methods

The consensus panel included 9 clinicians and scientific investigators from the United States and Europe. The decision of which clinicians and scientific investigators to invite was directed by the lead author (Konrad Bork), and was based on their HAE expertise as demonstrated by prior publications, involvement in key clinical trials, participation in previous guideline and recommendation projects, and roles in HAE-related professional societies. Among all authors, the median number of prior publications on HAE was 42. Additionally, all members of the panel had expressed a common interest in improving management and QoL for individuals with HAE. Prior to the consensus meeting, a systematic literature search of recent HAE guidelines and consensus papers was conducted to review the existing recommendations for (1) evaluating the severity of HAE attacks, (2) assessing HAE disease control, and (3) optimizing the long-term management of HAE. A systematic search of the PubMed database was performed, covering a 5-year publication period using the following search terms: hereditary angioedema, guideline, and consensus. The results of the systematic literature review were reviewed and edited by the lead author, with important guideline publications added outside of the 5-year time frame, which were cited by the author group as the most influential additional consensus references on the management of HAE. A first draft of the summary statements was drafted on the basis of the review results under the direction of the lead author and was sent to all panelists, along with the review. The panelists completed a survey to indicate their level of agreement with each summary statement on a Likert scale of 0 (strongly disagree) to 4 (strongly agree). Panelists were also given the opportunity to comment with additional recommendations for discussion regarding each statement. The consensus panel convened on February 29, 2020, and discussed the comments on the statements. At the end of the discussion, a new statement was drafted in the meeting, and the participants were surveyed using the aforementioned Likert score. Based on the Likert score, the panel declared whether they had reached consensus. One of the statements was revised after the meeting via e-mail to establish consensus. Throughout this review, all consensus recommendations are highlighted in bold text. All authors critically reviewed the information supporting the consensus statements and approved of their inclusion.

## Results

### Assessing severity

*Consensus Statement 1* HAE is a complex, life-threatening disease. PRO measures for assessing HAE attack severity and frequency are available and valuable tools, but a standardized approach for evaluation of attack severity in routine clinical practice is lacking. In addition, attack frequency and severity are insufficient markers of disease severity unless they are evaluated in the broader context of the effect on an individual patient’s QoL and ability to conduct activities of daily living.

Disease severity is difficult to determine for HAE [37]. Even in the presence of mild or no symptoms, HAE remains a serious and potentially life-threatening disorder [37]. All patients should prepare for a life-threatening attack regardless of attack frequency or previously experiencing a severe episode [14]. Therefore, existing guidelines recommend that all patients have immediate access to acute medication [4, 29, 30, 38]. Furthermore, severity can be influenced by multiple patient-specific factors including degree of disability and interference with daily activities [28, 37]. As such, the physical symptoms of HAE (eg, the frequency and severity of attacks) may not fully reflect the overall disease experience of the patient [28, 31].

Like overall disease severity, individual attack severity is also difficult to clinically quantify. Attacks are episodic, can be highly variable, and can occur simultaneously across multiple anatomical sites [2, 15, 39]. Attack severity is comprised of multiple factors including the location of the attack, the need for rescue medication, and the need for retreatment. Additionally, as severity is a subjective measure, the perceived severity of an attack may also be related to a patient’s experience and disruption in activities of daily living [29]. For example, an extremity attack with mild swelling may be considered severe by the patient if it significantly impacts their ability to work. The location of an attack is an important component of attack severity [29].

Abdominal swelling can cause mild to severe cramping pain with circulatory symptoms with or without vomiting and/or diarrhea; swelling of the extremities can cause discomfort and mobility limitations, and attacks involving the airway can be potentially fatal [12, 40, 41]. In a retrospective analysis of clinical case reports assessing the spatial patterns of HAE attacks in 221 patients with HAE-C1-INH, it was reported that attacks involving the skin (96%) and the abdomen (93%) are the most common [42]. While ≤ 1% of all attacks involve the larynx, more than 50% of patients with HAE will experience ≥ 1 laryngeal attack in their lifetime [42]. The need for acute treatment is another important factor in assessing attack severity. Guidelines recommend that all attacks should be considered for on-demand treatment and those potentially involving the upper airways should be treated as early as possible to prevent suffocation [30]. Decisions to treat other attacks may be left to the individual patient, who may consider perceived treatment burden, response to therapy, and whether the swelling is likely to result in disability [29]. Not all patients will adequately respond to a single dose of acute treatment; some may require repeated dosing to achieve symptom control [29, 43].

One approach to monitoring and assessing attack severity is for patients to keep a record of their attacks in a diary in order to capture a description of the attack, any treatment used, and the response to treatment [29]. These diaries can be helpful for capturing real-time information on attacks and provide useful information on the use of acute medications; however, long-term adherence to daily reporting can be low in the clinical setting and add burden to a patient’s life.

Although validated tools to assess HAE attack severity in routine clinical practice are limited, several PRO measures of attack severity have been used in clinical trials to quantify the effectiveness of acute HAE therapies [44–46]. Examples of PRO measures used in clinical trials include the visual analog scale (VAS), composite scales such as the mean severity complex score (MSCS) and treatment outcome score (TOS), and other Likert-type scales [29, 37]. The VAS instrument asks patients to indicate the severity of HAE symptoms on a continuous 100-mm scale, where 0 mm indicates “no symptoms” and 100 mm indicates “extremely disabling” [44, 47]. VAS scores are quick and easy to use and may be applied to the evaluation of both general attack severity and specific attack symptoms, but they do not provide a composite score [37, 48]. The MSCS evaluates the mean global symptom severity at a specified time point (eg, following the administration of study drug) [37, 49]. The MSCS measures 2 components: the anatomical site of each symptom (symptoms complex) and the severity of each symptoms complex (on a scale of 0 to 3) [49]. Higher scores indicate more severe symptoms [49]. The TOS evaluates a patient’s recollection of changes in symptom severity in response to treatment [49]. The TOS is comprised of 3 components: the anatomical site of each symptom (symptoms complex), the severity of each symptoms complex at baseline (on a scale of 1 to 3), and the response assessment at 4 and 24 h post dosing (on a scale of − 100 to 100) [49]. Higher scores indicate a more significant improvement in symptoms from baseline following treatment. Although both the MSCS and TOS consider all symptoms experienced, allowing for the assessment of variable swelling patterns commonly observed during HAE attacks, these tools are more complex and less likely to be used in routine practice [37]. Other Likert-type severity scales have been used in clinical trials for targeted HAE therapies; however, a standardized assessment approach is lacking [29, 35].

### Evaluating disease burden

*Consensus Statement 2* HAE may be associated with significant disease burden, which interferes with a patient’s QoL both during and between attacks. Determination of HAE disease burden includes assessment of frequency and severity of attacks as well as effects on QoL. The assessment of disease burden can be used to identify targets for improvement and assess treatment outcomes. QoL assessments should be individualized for each patient and at minimum, they should address the interference of HAE with work, school, social, family, and physical activity, along with access to and burden of HAE treatment.

**Fig. 1:**
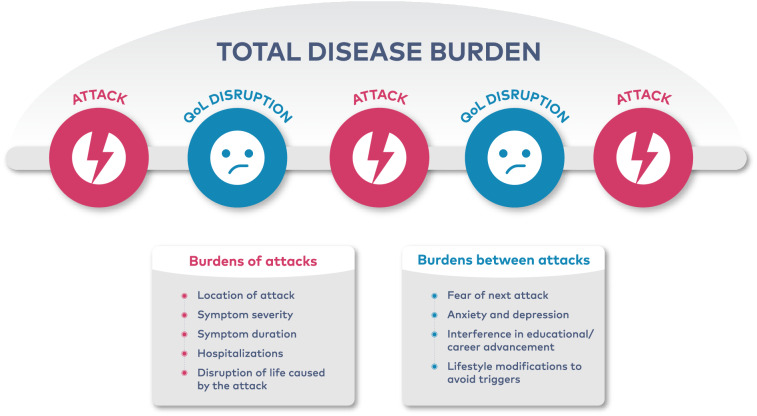
Assessment of disease burden. QoL, quality of life

Disease burden is a larger measure than severity and includes the frequency and severity of attacks as well as detriments to QoL suffered during and between attacks, including interference with activities of daily living, and heightened emotional distress (Fig. 1). Patients with HAE may experience significant fear or anxiety in anticipation of their next attack or make lifestyle modifications in an effort to reduce the likelihood of an attack [16, 17, 19, 27]. In the HAE burden of illness study (HAE-BOIS) in Europe, a cross-sectional survey assessing the real-world experience of patients with HAE, patients reported considerable interference in career and educational advancement due to absenteeism [18]. Additionally, survey respondents reported significant impairment caused by pain or discomfort and depression or anxiety both during and between attacks [50]. In a Danish cohort study, more than one-half of patients felt that HAE had a significant psychological impact on their lives and restricted their physical activities [17]. As the clinical expression of HAE is highly variable, the effects of the disease on patient experience are also highly variable [15]. Therefore, assessment of disease burden should be tailored to each patient. Accurate assessment of disease burden can help identify areas for improvement and optimize treatment.

Several validated tools for the assessment of disease burden are available for use in patients with HAE (Table 1). Two PRO measures are available to assess how active the disease is at a given point in time (disease activity) [37, 51, 52]. The angioedema activity score (AAS) has been used to assess all forms of recurrent angioedema, including hereditary angioedema [27, 51]. With the AAS, patients prospectively record the occurrence of HAE symptoms over a 24-h period [51]. If symptoms occur, patients complete 5 additional questions regarding the episode, including level of discomfort, effect on daily activities, and overall perceived severity [37, 53]. Patient data collected over a period of at least 4 consecutive weeks are combined to obtain a measure of disease activity [53]. While the AAS has good internal consistency and brevity, its prospective nature can result in challenges with compliance [51, 53]. The HAE activity score (HAE-AS) is a PRO measure developed specifically to assess HAE-C1-INH [52]. This retrospective assessment consists of 12 items, of which 7 pertain to attacks occurring over the previous 6 months, and 5 pertain to emergency visits, psychological status, days of school/work missed, impairment in work/activities due to pain, and general health [52]. The HAE-AS retrospectively assesses activity over a longer period of time than the AAS and can therefore account for variation in attacks and impact on daily life; however, it requires patients to accurately recall disease experiences over the previous 6 months [52].

**Table 1:** Quality of Life and PRO assessments to evaluate disease burden and control

Assessment	Application	Number of items	Recall period(s)	Advantages	Disadvantages
Angioedema Activity Score (AAS) [51]	Recurrent angioedema	5	24 h	Brief Good internal consistency	Prospective nature limits compliance
HAE Activity Score (HAE-AS) [52]	HAE-C1-INH	12	1 month and 6 months	Allows for assessment of attack variability over time	Requires accurate recall over 6 months
Angioedema Quality of Life (AE-QoL) Questionnaire [57]	Recurrent angioedema	17	4 weeks	Good psychometric properties Good internal consistency	Time-consuming Not HAE specific
Hereditary angioedema quality of life (HAE-QoL) [61]	HAE-C1-INH	25	6 months	HAE specific Good internalconsistency	Time-consuming Requires accurate recall over 6 months
United States Hereditary Angioedema Association Quality of Life (HAEA-QoL) Survey [65]	HAE-C1-INH	27	NR	NR	Validation studies are needed
36-Item Short-Form Health Survey (SF-36) [54, 93]	Generic health status	36	1 week and 4 weeks	Useful for comparisons	Less specific Low sensitivity
EuroQol 5-Dimensions Survey (EQ-5D) [54, 94]	Generic health status	5	No recall period	Useful for comparisons Easy to administer	Less specific Low sensitivity
Angioedema Control Test (AECT) [59]	Recurrent angioedema	4	4 weeks and 3 months	Brief Simple scoring	Validation studies are needed

HAE, hereditary angioedema; HAE-C1-INH, HAE with C1-esterase inhibitor deficiency; NR, not reported; PRO, patient-reported outcome

One of the best ways to assess disease burden is to evaluate a patient’s QoL [54]. Key considerations in the assessment of QoL include the frequency and severity of attacks, anxiety and fear, activities of daily living and productivity, social and family burdens, physical activity, frequency of hospital visits, attack triggers, and comorbid conditions. Additional factors such as treatment burden and access to acute therapy are critical to the assessment of QoL, as they can amplify fear and anxiety about attacks. A recommended list of questions asked by physicians to assess a patient’s overall disease burden are listed in Table 2. It is also important to understand nonverbal cues, particularly when assessing the psychological burden of the disease.

**Table 2:** Recommended list of questions to assess burden of disease in patients with HAE

Are there any activities that you avoid because of your HAE?
How often do you experience HAE attacks?
How would you describe the severity of your HAE attacks? (0 = no impairment; 4 = complete disablement)
How often does HAE cause you to miss work, school, or activities at home?
How often do you have to use acute rescue medication for each HAE attack and do you feel that you respond well?
What is the average time from attack onset to treatment administration? Time to initial symptom relief? Time to complete resolution of symptoms?
Have you had any changes in life status that may affect the activity of your HAE?
How often do you experience fear/anxiety/depression associated with your HAE?
Have you had any difficulties accessing or administering your acute or prophylactic HAE treatment?
To what extent has HAE interfered with your social life, family, relationships, or physical activities?
How often have you had to visit the hospital for an HAE attack?
Have you made any lifestyle modifications in an effort to avoid attack triggers?

HAE, hereditary angioedema

Several different tools have been developed for the purpose of assessing QoL including generic instruments such as the 36-item Short-Form Health Survey (SF-36) and the EuroQoL 5-dimensions survey (EQ-5D), and condition-specific instruments such as the angioedema quality of life (AE-QoL), hereditary angioedema quality of life (HAE-QoL), and United States Hereditary Angioedema Association quality of life (HAEA-QoL) assessments (Table 1) [37, 54]. Generic instruments have been used in survey studies and clinical trials to compare QoL measures with healthy populations or different disease states, and to estimate health utility and evaluate the effects of a study drug on patients’ QoL [21–27, 50, 55, 56]. For example, in a cross-sectional survey study of Puerto Rican patients with HAE-C1-INH, results from the generic SF-36 showed that ≥ 50% of patients scored lower than the normative US population in all elements of the physical and mental domains, demonstrating reductions in QoL [22]. While useful for comparisons, generic assessments are less specific and often have lower sensitivity for measuring disease-specific components [37, 53].

The AE-QoL assessment can be used to evaluate QoL in patients with recurrent angioedema [57]. It consists of 17 items grouped in 4 domains (functioning, fatigue/mood, fears/shame, and food) rated over the previous 4 weeks [57]. The AE-QoL has been used in some randomized clinical trials for HAE and in clinical practice; however, it can be time-consuming to administer and analyze, and is not specific to HAE [58–60]. The HAE-QoL questionnaire is specific for patients with HAE-C1-INH and consists of 25 items grouped into 7 domains: treatment difficulties, physical functioning and health, disease-related stigma, emotional roles and social functioning, concerns about offspring, perceived control over illness, and mental health [54, 61]. Patients complete the questionnaire based on perceived QoL over the previous 6 months [37]. Although the HAE-QoL has been used in clinical practice [62, 63], like the AE-QoL, it can be time-consuming to administer and analyze. Currently in development, the HAEA-QoL is a 27-item questionnaire designed to assess QoL in patients with HAE-C1-INH based on management guidelines in the United States [64]. The 27 items are divided into 2 domains: an emotional and social well-being “feelings” domain, and an HAE-specific “concerns” domain [65]. Additional validation studies of this tool are ongoing [65].

While existing instruments provide valuable measures of QoL, they may not be sensitive enough to accurately reflect changes in patients’ QoL in and outside of clinical trials. For example, in a post hoc analysis of the phase 3 COMPACT trial, the mean change in EQ-5D scores between 60 IU/kg of SC C1-INH and placebo was small (mean treatment difference, 0.04 [95% confidence interval, − 0.04 to 0.11]) and not suggestive of a treatment benefit [66]; however, in the primary analysis, 60 IU/kg of SC C1-INH was associated with an 84% mean reduction in attacks relative to placebo [34]. Conversely, in the phase 3 HELP trial assessing changes in QoL with prophylactic SC lanadelumab, significant reductions relative to placebo in total and specific QoL domain scores were observed using the AE-QoL questionnaire (*P* < 0.01 for all) [58]. A possible limitation of existing QoL assessments is that they do not consider the effects of the treatment (eg, convenience, side effects). In some cases, the treatment may present a considerable burden affecting QoL. While QoL scores may not allow comprehensive assessment of disease burden, they provide important information that should be considered along with patient interviews and other disease assessments.

Although tools that assess disease activity and QoL are valuable measures of HAE disease burden, they do not assess the level of control that patients have of their disease at a specific time point [37]. Disease control is a particularly important measure for chronic diseases because it can support treatment decisions and help assess patient responses to prophylactic therapy [37, 59]. The angioedema control test (AECT) is a 4-item PRO measure developed to retrospectively assess disease control over time in patients with recurrent angioedema [59]. The 4 items of the AECT assess the signs and symptoms, impact, effectiveness of treatment, and unpredictability [59]. There are 2 versions of the AECT, one with a recall period of 4 weeks, and another with a recall period of 3 months [59, 67]. The retrospective approach, brevity, and simple scoring of the AECT allow for its application in routine clinical practice and clinical trials; however, further validation studies in broader populations are needed to characterize its reliability [59].

### Long-term clinical management of HAE

*Consensus Statement* 3 Management of HAE requires comprehensive treatment tailored to the individual patient based on disease burden and individual circumstances. Every patient with HAE should have immediate access to acute treatment and short-term prophylaxis as required. Long-term prophylaxis (LTP) should be discussed with every patient and should involve shared decision-making between the patient and physician, along with routine monitoring and adjustment of the management plan as needed. Advances in LTP therapies allow patients to achieve minimal attack burden with reduced disease and treatment burden.

**Fig. 2:**
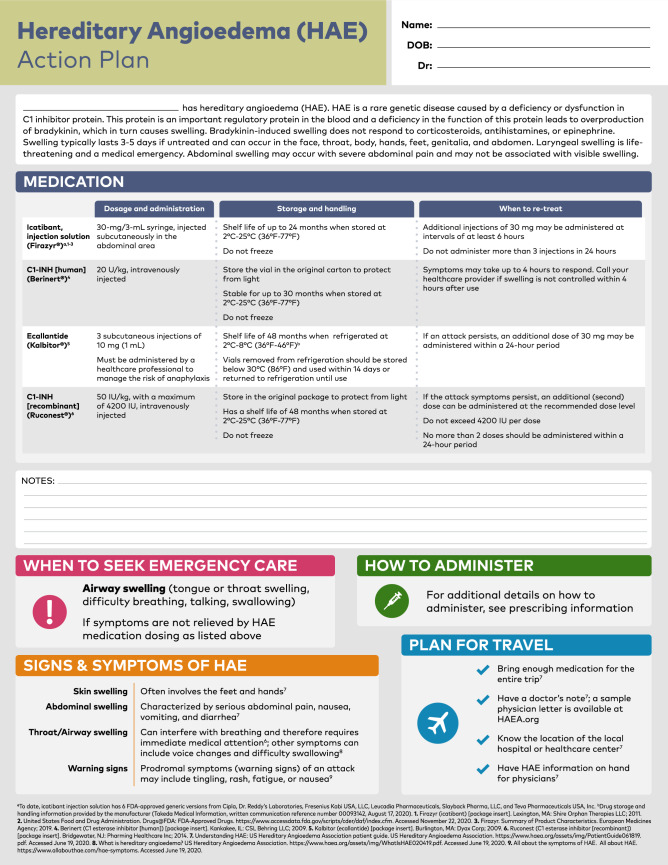
On-demand treatment action plan. HAE, hereditary angioedema

Due to the chronic and unpredictable nature of HAE, optimal long-term management involves an individualized treatment plan developed by the physician and each patient, and may include both acute and preventative measures [30]. To minimize morbidity and prevent mortality from an HAE attack, existing guidelines recommend that all patients have access to at least 2 standard doses of acute medication to treat angioedema symptoms when they occur [29, 38]. An effective acute treatment plan should contain clear instructions on how to best use medications to treat attacks, including how the treatment will be administered (eg, self-administration) and how to determine whether additional dosing or medical attention is needed (Fig. 2) [29, 30].

Available acute medications include intravenous (IV) plasma-derived C1-INH (Berinert®, CSL Behring LLC, King of Prussia, PA), IV recombinant C1-INH (Ruconest®, Pharming Healthcare Inc, Bridgewater, NJ), SC bradykinin B2-receptor antagonist (icatibant [Firazyr®], Shire Orphan Therapies LLC, Lexington, MA), and SC plasma kallikrein inhibitor (ecallantide [Kalbitor®], Shire US Inc, Lexington, MA) [68]. Additionally, six generic versions of SC icatibant have been approved by the US Food and Drug Administration (FDA) to date [69–74]. Plasma-derived and recombinant C1-INH concentrates are administered as IV infusions of 20 U/kg and 50 U/kg, respectively [75, 76]. While both treatments have favorable safety profiles, potential adverse events (AEs) include the very rare risk for anaphylaxis and possible, but unlikely thromboembolic events [75, 76]. Ecallantide is administered in 3 SC injections of 10 mg (1 mL) [77]. The primary safety concern with ecallantide treatment is the risk for anaphylaxis [78]. Icatibant is administered as a 30-mg SC injection in the abdominal area [79]. In clinical trials, the most commonly reported AEs associated with icatibant were injection site reactions, reported in 95% to 98% of patients across treatment populations [79, 80].

Preventative measures such as management of suspected triggers and prophylactic therapy may also be discussed with patients as part of a long-term management plan. Current guidelines recommend that all patients with HAE should be educated about possible triggers, which may induce HAE attacks [29, 30, 38]. While most attacks occur spontaneously, it is important to search for potential exacerbating triggers and assess the viability of lifestyle or medical modifications to avoid them. Examples of triggers include physical trauma, psychological stress, angiotension-converting enzyme inhibitors, estrogen-containing medications, and infection. Triggers can have varied effects on HAE disease, and what may be a triggering factor for one patient may not affect another. Attempts to avoid and modify triggers should not preclude prophylactic treatment [30]. Short-term prophylaxis should be discussed and recommended as a preventative measure before surgical or invasive dental procedures or before stressful life events expected to trigger an attack [28, 29]. In a retrospective study assessing the risk of swelling following tooth extraction in patients with HAE, 21.5% of patients not receiving prophylaxis experienced postprocedural facial or laryngeal edema compared with 12.5% of patients receiving short-term prophylaxis [81]. Additional studies are needed to understand the ongoing requirement for short-term prophylaxis in patients treated with newer, highly effective long-term prophylaxis options [30].

Long-term prophylaxis is intended to lessen the disease burden for patients by reducing the frequency and severity of attacks and restoring a normal QoL. Therapies available for long-term prophylaxis of HAE attacks are included in Table 3. Antifibrinolytics are not approved by the US FDA for use in patients with HAE and recent evidence-based guidelines do not recommend use for long-term prophylaxis in HAE-C1-INH due to inferior efficacy data [30]. However, they have been used for prophylaxis of HAE attacks in regions/countries where other, more effective HAE therapies are not available [28, 30]. Despite their effectiveness, oral androgens have numerous side effects such as weight gain, hyperlipidemia, virilization in women, liver toxicity, acne, and menstrual abnormalities that limit tolerability and lead to contraindications in certain populations [30, 82–84]. IV and SC formulations of C1-INH replacement therapy are also available for long-term prophylaxis. Twice-weekly IV C1-INH (1000 U) was approved in the United States in 2008 for routine prophylaxis in adults and adolescents based on the results of a phase 3 clinical trial demonstrating a significant reduction in normalized attack rates over 12 weeks relative to placebo (C1-INH 1000 U: 6.26 attacks, n = 11; placebo: 12.73 attacks, n = 11; *P* < 0.001) [28, 35]. In 2018, approval of twice-weekly IV C1-INH was extended to pediatric patients based on a phase 3 trial in patients with HAE aged 7–11 (n = 12) [85, 86]. The 500-U dose of IV C1-INH was shown to reduce the monthly number of attacks by 71% over 12 weeks compared with baseline attack rates (mean, 1.15 vs 3.72 attacks/month) [86]. In 2017, a SC formulation of C1-INH was approved in the United States for routine prophylaxis in adolescents and adults [28, 87]. Approval was based on results of the phase 3 COMPACT trial in which patients receiving 60 IU/kg of C1-INH twice weekly (n = 43) had a significant mean reduction in attacks per month versus placebo (n = 42) through 16 weeks of treatment (mean, 0.52 vs 4.03 attacks/month; *P* < 0.001) [34].

**Table 3:** Summary of prophylactic treatments

Generic name (trade name)	Dosage	Mechanism	Approval status	Efficacy^a^	Potential adverse events
Plasma-derived C1-INH (Cinryze®) [35, 85]	Adults and adolescents: 1000 U IV every 3 or 4 days Children: 500 U IV every 3 or 4 days	C1-INH	Approved for prophylaxis in adults, adolescents, and pediatric patients aged ≥ 6 years	Normalized attack rate was 6.26 attacks/12 weeks compared with 12.73 with placebo	Rash, lightheadedness, fever
Plasma-derived C1-INH (Haegarda®) [34, 87]	60 IU/kg SC twice weekly	C1-INH	Approved for prophylaxis in adults and adolescents	Mean number of attacks/month was 0.52 compared with 4.03 with placebo	Injection site reaction, hypersensitivity, nasopharyngitis, dizziness
Lanadelumab (Takhzyro®) [33, 88]	300 mg SC Q2W Dosing Q4W may be considered in patients with favorable response after 6 months	Plasma kallikrein inhibitor (monoclonal antibody)	Approved for prophylaxis in adults and adolescents	Mean number of attacks/month was 0.26 compared with 1.97 with placebo	Injection site reaction, dizziness
Berotralstat (Orladeyo®) [36, 89]	150 mg oral QD	Plasma kallikrein inhibitor	Approved for prophylaxis in adults and pediatric patients aged ≥ 12 years	Mean attack rate of 1.31 attacks/month compared with 2.35 attacks/month with placebo	Abdominal pain, vomiting, diarrhea, back pain
Danazol (Danocrine®) [95, 96]	200 mg oral QD Dose should be titrated to the lowest clinically effective dose	17-alpha-alkylated androgen, Mechanism unknown	Approved for the prevention of attacks of angioedema in adults	Attacks occurred in 2.2% of danazol courses compared with 93.6% of placebo courses	Weight gain, virilization, acne, menstrual abnormalities, muscle pains, headaches, fatigue, nausea, hypertension
Tranexamic acid (Lysteda™) [28, 97, 98]	30–50 mg/kg QD	Antifibrinolytic	Not FDA approved	Of the 12 patients with C1-INH treated with tranexamic acid over 6 months, 6 experienced no reduction in HAE attacks, 3 experienced a moderate reduction, and 3 experienced a large reduction (> 75%)	Gastrointestinal events, myalgia/creatine kinase elevation, risk of thrombosis

C1-INH, C1-esterase inhibitor; FDA, Food and Drug Administration; IV, intravenous; NDA, new drug application; Q2W, every 2 weeks; Q4W, every 4 weeks; QD, once daily; SC, subcutaneous. ^a^Differences in trial design and populations limit cross-trial comparisons

In 2018, SC lanadelumab, a monoclonal antibody to plasma kallikrein, was approved for routine prophylaxis in adults and adolescents [88]. Approval was based on a phase 3 HELP trial demonstrating that 300 mg of SC lanadelumab twice monthly (n = 27) significantly reduced mean monthly HAE attack rates versus placebo (n = 41) over 26 weeks (mean, 0.26 vs 1.97 attacks/month; *P* < 0.001) [33].

In 2020, berotralstat (BCX7353), an oral, once-daily inhibitor of plasma kallikrein was approved in the United States and Japan for prophylaxis to prevent attacks of HAE in adults and pediatric patients 12 years and older [89]. Approval was based on results of the phase 3 APeX-2 trial showing that berotralstat 150 mg reduced monthly attack rates over 24 weeks (1.31 attacks/month; n = 40) compared with placebo (2.35 attacks/month; n = 40; *P* < 0.001) [36]. Berotralstat is the first targeted, once-daily, oral medication approved for prophylaxis of HAE attacks.

**Fig. 3:**
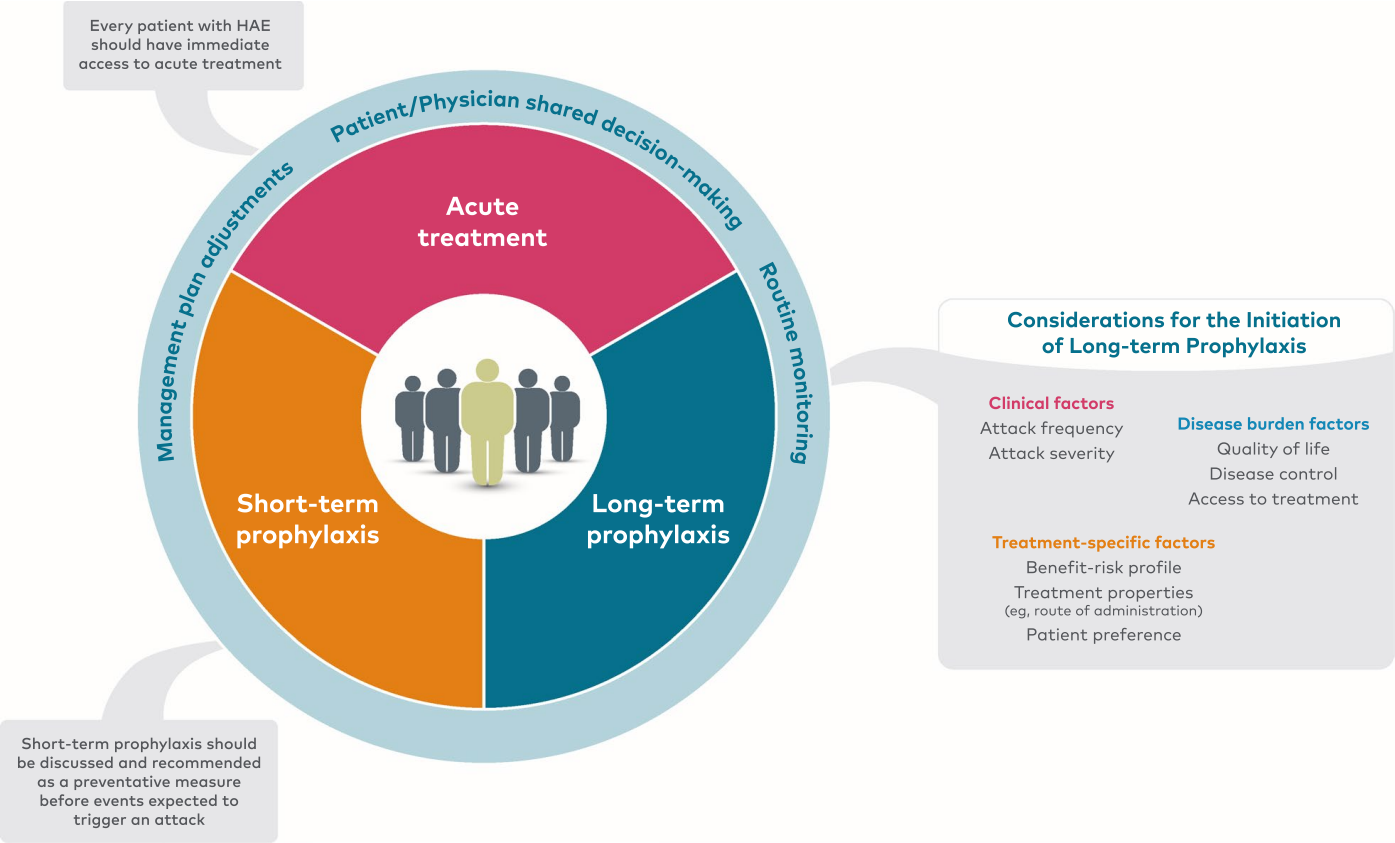
Considerations for initiating long-term prophylaxis. HAE, hereditary angioedema

The options for long-term prophylaxis should be discussed with every patient and should consider clinical factors such as attack frequency and severity, as well as components of disease burden including patient QoL, disease control, and access to treatment (Fig. 3) [29, 30]. Moreover, with the availability of newer prophylactic options, decisions regarding the initiation of long-term prophylaxis should also consider the benefit-risk profiles and treatment properties of available therapies with the goal of improving patient outcomes and reducing treatment burden [28, 90]. For example, compared with IV prophylactic therapy, newer SC and oral therapies may be considered to reduce treatment burden for patients with venous access problems or for those uncomfortable with administering IV infusions. In a survey study of patients with HAE using IV long-term prophylaxis, 62% of respondents who used a peripheral vein to administer treatment had reported difficulties finding usable veins or administering the infusion [91]. In another survey study of patients with HAE evaluating the comfort of self-administering medication, only 51% of respondents reported that they would be comfortable administering IV treatment [92]. Additionally, newer prophylactic therapies that have less frequent or simpler dosing regimens may reduce the time dedicated to and discomfort associated with treatment and thereby, treatment burden.

As with other chronic diseases, there is desire for a precision medicine approach (ie, choosing the right medicine for the patient). Although data on precision medicine in HAE are lacking, it is known that individual patients respond differently to various prophylactic options and there are currently no biomarkers to predict response (ie, reduction or elimination of HAE attacks). Therefore, individualized treatment plans may be developed based on measurable patient-specific factors and preferences. Indication of successful prophylactic therapy would be a significant reduction or elimination of HAE attacks. However, as the goal of long-term prophylaxis is to reduce the overall burden of disease for patients, QoL should also be assessed [30]. For example, the ability to perform activities that would have previously been avoided or caused an attack could be an indicator of successful use of prophylaxis. Additionally, because prophylaxis of HAE attacks requires long-term treatment, adherence is an important measure of successful therapy [30].

As disease activity, disease burden, and other factors can vary over time, patient treatment plans should be monitored and reviewed continually [29]. Guidelines recommend that routine evaluation should include the frequency and severity of symptoms, and the efficacy and frequency of acute medication use [29]. The option for long-term prophylaxis should be examined at each visit, especially if anticipated life or health events may make a patient more susceptible to an increase in disease activity [30]. Patients who are already on long-term prophylaxis should also be regularly assessed for efficacy, safety, and adherence [30]. It is suggested that physicians should assess patients every 6 to 12 months; however, it may be necessary to have more frequent visits for new patients or for patients changing their treatment plan [29].

## Conclusions

HAE is a variable, severe, and life-threatening condition with significant disease burden. Determining and developing an optimal treatment path for patients involves consideration of multiple interrelated components including frequency and severity of attacks, disease burden, and disease control. Some validated tools are available to assess these components; however, improvements are needed to expand their clinical utility. Evaluation of HAE disease burden should include assessment of frequency and severity of attacks as well as effects on QoL. Key considerations in the assessment of QoL include interference of HAE with work, school, social, family, and physical activities, along with access to and burden of HAE treatment. Significant progress has been made to develop HAE-specific therapies with improved efficacy/safety profiles and differing mechanisms of action and routes of administration. These additional options allow for a tailored treatment approach, taking into consideration patients’ preferences and treatment goals, as well as specific medication profiles. Despite global differences in treatment patterns, these consensus statements should build on existing HAE guidelines and provide useful assessment approaches for all clinicians treating patients with HAE.

## Data Availability

Not applicable.

## Abbreviations

AAS: Angioedema activity score
AE: Adverse event
AECT: Angioedema control test
AE-QoL: Angioedema quality of life
C1-INH: C1-esterase inhibitor
EQ-5D: EuroQol 5-dimensions survey
FDA: Food and Drug Administration
HAE-AS: Hereditary angioedema activity score
HAE-BOIS: Hereditary angioedema burden of illness study
HAE: Hereditary angioedema
HAE-C1-INH: HAE with C1-esterase inhibitor deficiency
HAE-QoL: Hereditary angioedema quality of life
HAEA-QoL: Hereditary Angioedema Association quality of life
IV: Intravenous
LTP: Long-term prophylaxis
MSCS: Mean symptom complex severity
PRO: Patient-reported outcome
QoL: Quality of life
SC: Subcutaneous
SF-36: 36-item short-form health survey
TOS: Treatment outcome score
VAS: Visual analog scale
